# Comparative Evaluation of Two Different Preparation Designs and Materials on the Fracture Resistance of Laminate Veneers: An In Vitro Study

**DOI:** 10.7759/cureus.66811

**Published:** 2024-08-13

**Authors:** Pranjal S Soni, Pronob Sanyal

**Affiliations:** 1 Department of Prosthodontics and Crown and Bridge, School of Dental Sciences, Krishna Vishwa Vidyapeeth (Deemed to be University), Karad, IND

**Keywords:** instron universal testing machine, tooth preparation for laminate veneer, fracture resistance, upcera, cerec tessera, porcelain laminate veneers

## Abstract

Introduction

Laminate veneers are thin porcelain or composite resin shells bonded to the front of teeth for cosmetic enhancement. The design of the tooth preparation is crucial; it involves minimal enamel removal to ensure a proper fit and strong bond. This careful preparation increases the veneers' fracture resistance by preserving tooth structure and evenly distributing stress, ensuring durability and natural appearance.

Aim

Our aim was to determine the fracture resistance of two preparation designs with two different materials for the fabrication of laminate veneers.

Materials and methods

An in vitro study was conducted on 40 laminate veneers divided into two groups based on the material used: Group 1 (Cerec Tessera (Dentsply Sirona, Charlotte, NC, USA)) and Group 2 (Upcera, Shenzhen, China). Each group was further divided into two sub-groups based on tooth preparation designs: butt joint and feather edge. The fracture resistance of these veneers following thermocycling was determined, and the mode of fracture was evaluated using a stereomicroscope.

Results

Feather edge preparation with Cerec Tessera laminate veneer restoration demonstrated the highest fracture resistance at 111.5 N, followed by butt joint preparation with Cerec Tessera laminate veneer restoration, which showed a fracture resistance of 90.8 N. The study identified laminate fractures as the most common type of fracture observed in the veneer restorations, followed by cement interface fractures.

Conclusion

The study suggests that the feather edge preparation design contributed to higher fracture resistance compared to the butt joint design. This finding aligns with the idea that modifying the preparation design can have a significant impact on the overall strength and durability of veneer restorations. The use of Cerec Tessera, especially in combination with the feather edge design, yielded the maximum fracture resistance.

## Introduction

Porcelain veneers are useful for treating a wide range of aesthetic issues that patients may experience, such as diastema closure, tooth discolorations like hypocalcifications, dental fluorosis, and tetracycline stains; the correction of hypoplastic enamel defects like amelogenesis imperfecta and dentinogenesis imperfecta; and the correction of malformed teeth, such as peg lateral incisors [1].

There is a wide variety of ceramic materials available, each offering unique benefits based on the amount of tooth structure remaining after preparation and the material's inherent ability to withstand occlusal forces. There is no consensus on the specific type of porcelain veneer preparation necessary to ensure the restoration's durability and strength over time. After 25 years of ongoing research, several practitioners have recommended the following four preparation designs: facial window preparation, butt joint preparation, palatal chamfer preparation, and feather edge preparations that provide an incisal edge in porcelain and enamel [2]. Using various ceramic materials, laminate veneers can enhance the aesthetics of anterior teeth. Ceramics can be categorized into three groups based on the following factors: composition, sinterization temperature, and production process.

Ceramics are divided into several categories based on their composition, such as glass-based ceramics, alumina-based ceramics, and zirconia-based ceramics [3]. Contemporary cement systems use a dual-cure resin-based option to aid the cementation process. However, while cementation may be the most important component of the procedure, it is frequently cited as the main cause of restoration failures within the first 24 to 48 hours after delivery due to factors like moisture contamination, voids at the margins, inadequate bonding material, and other issues. While everyone agrees that there are multiple preparation types that can be used in various clinical settings, determining which one allows for the optimal degree of form, function, longevity, and aesthetics remains a challenge. For patients who meet the requirements and are willing to undergo the treatment, we may be able to recommend one optimal therapeutic approach after conducting empirical testing and interpreting the findings. This would enable our practitioners to apply a standard treatment approach that has been shown to be dependable and provides our patients with the desired aesthetics. Accordingly, this study was conducted to examine the effect of two different preparation designs and materials on the fracture resistance of laminate veneers after thermocycling.

## Materials and methods

**Figure 1:**
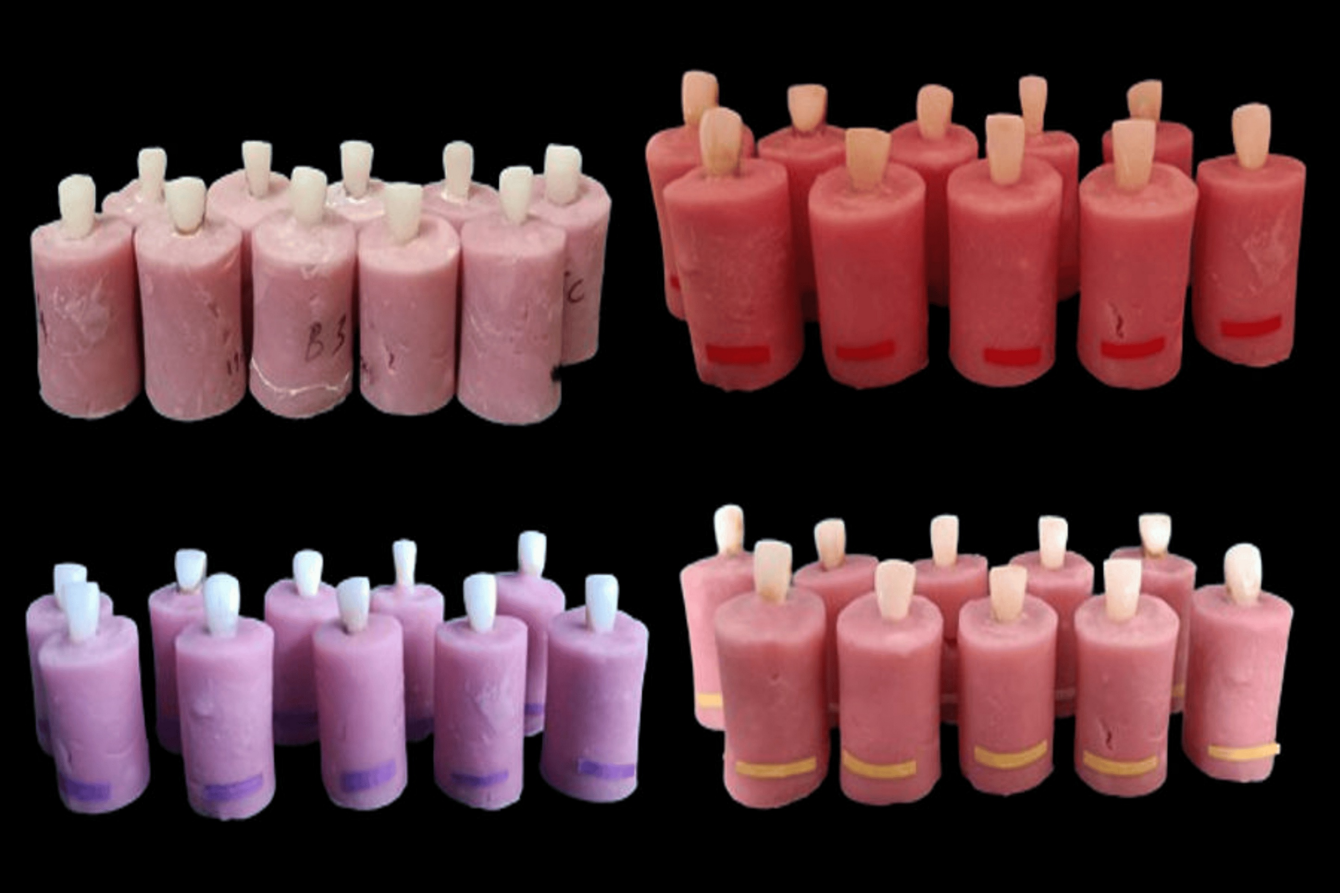
Group 1 A1 Cerec Tessera - Butt Joint (PINK), Group 1 B1 Cerec Tessera - Feather Edge (RED), Group 2 A2 Upcera - Butt Joint (PURPLE), Group 2 B2 Upcera- Feather edge (YELLOW)

A sample size of 40 dental veneers was determined by the software G*Power 3.1.9.2 (Heinrich Heine University of Düsseldorf, Germany) and divided into four subgroups of 10 samples each. Forty non-carious extracted maxillary central incisors were collected and cleaned of gross debris in a sodium hypochlorite solution (1:10) and kept in distilled water (Vitzee Chemicals, Pune, India) to maintain hydration. Block samples were created using a silicone mold which was 20 mm long, 20 mm wide, and 63.5 mm in height, prepared according to ISO standardization. The split mold was filled with auto-polymerized acrylic resin material that had been mixed in accordance with the manufacturer's instructions, and the central incisors were positioned in the middle of the blocks, vertically and perpendicular to the base. After polymerization, the acrylic blocks were removed from the mold and labeled into the respective groups: Group 1 A1 Cerec Tessera - Butt Joint (PINK), Group 1 B1 Cerec Tessera - Feather Edge (RED), Group 2 A2 Upcera - Butt Joint (PURPLE), and Group 2 B2 Upcera - Feather Edge (YELLOW) (Figure 1).

**Figure 2:**
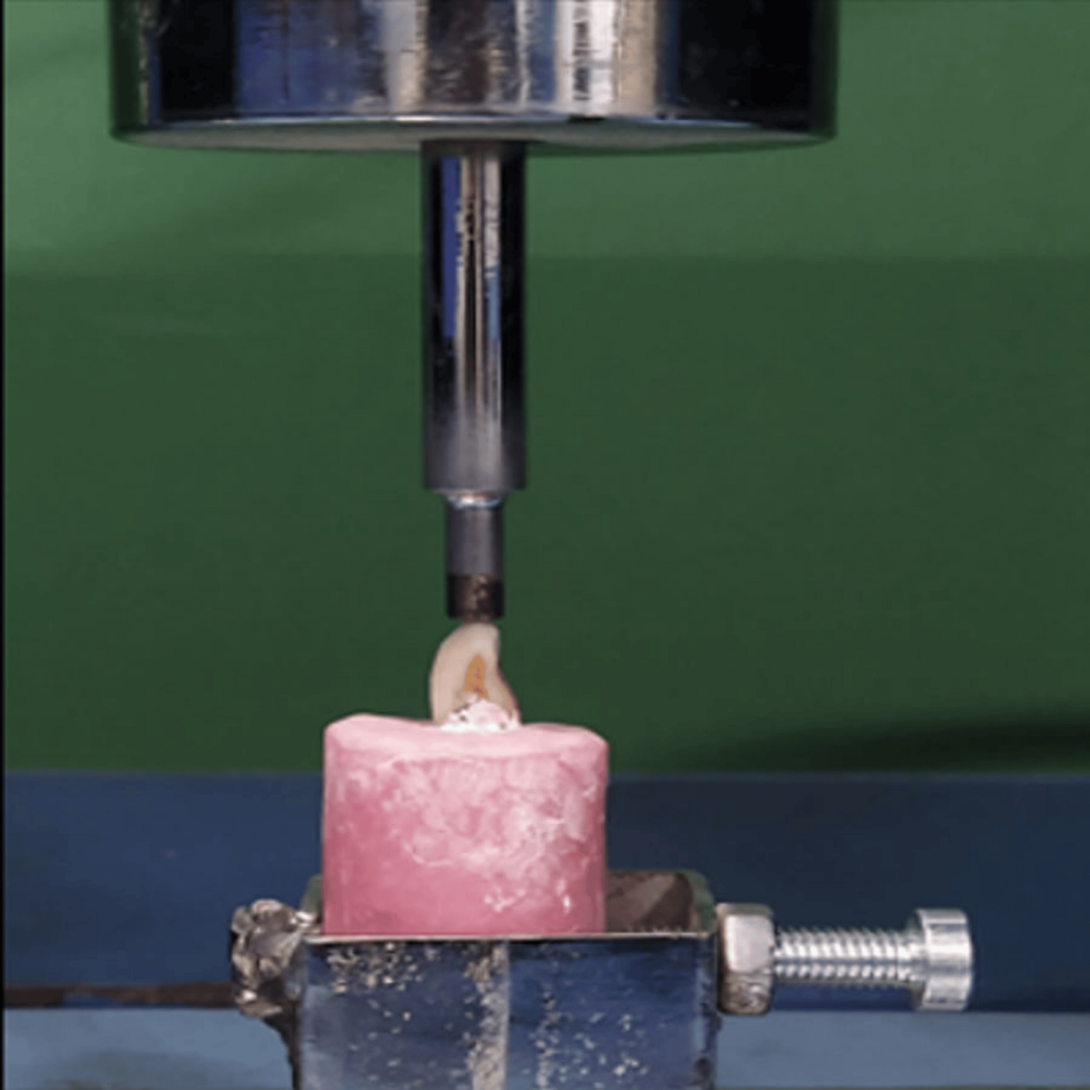
Mounted sample on Universal Testing Machine

A single operator carried out the tooth preparation of the laminate veneers on the abutment teeth samples to maintain uniformity. Twenty central incisors with butt joint preparation, including incisal bevel preparation and 1 mm incisal reduction, were prepared, and 20 central incisors with feather-edged preparation technique, where the preparation extends to the incisal edge with no reduction of the incisal edge, were also prepared. The prepared central incisors were scanned using computer-aided design (CAD) software. The STL files were transferred to computer-aided manufacturing (CAM) software, where the veneers were milled from Cerec Tessera (Dentsply Sirona, Charlotte, NC, USA) Ingots and Upcera (Shenzhen, China) Ingots. The veneers were then luted to the prepared teeth using self-adhesive resin cement (Panavia SA Cement Universal; Kuraray Noritake Dental, Tokyo, Japan). The cement was mixed according to the manufacturer’s recommendations, the veneers were seated, and the cement was allowed to set. Excess cement was wiped off, and a final curing of 10 seconds was performed. The pressure applied during cementation was by hand (fingertip) without any additional device, applied by the same operator. The specimens were stored in a water bath at 37°C for one week, after which they were subjected to thermocycling for 10,000 cycles between 5°C and 55°C, with a dwell time of 30 seconds. This simulated the intra-oral performance of the crowns for one year. Each thermocycled specimen was then mounted in a universal testing machine (Figure 2).

**Figure 3:**
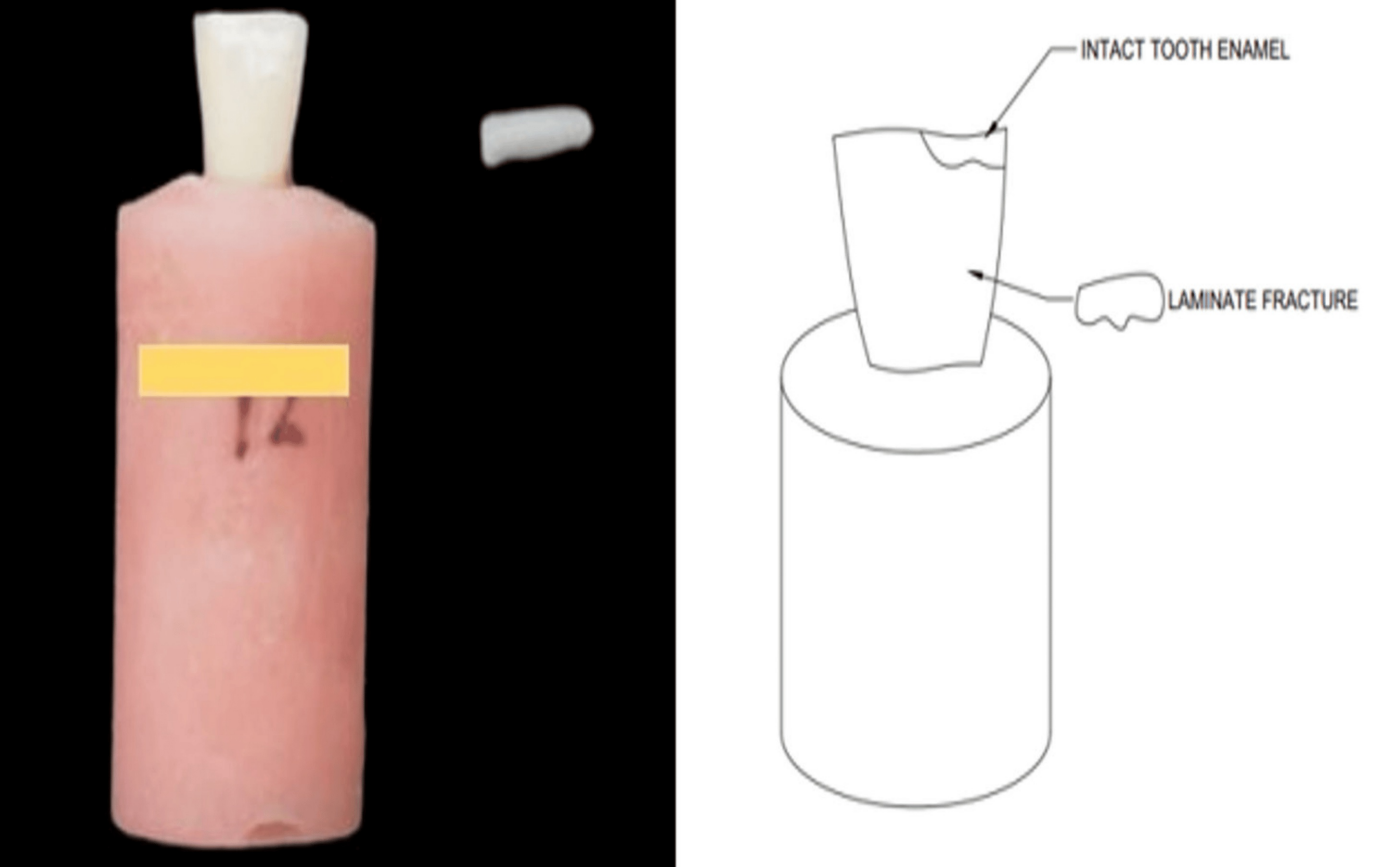
Laminate fracture explained with schematic diagram. Image credit: Pranjal S Soni

**Figure 4:**
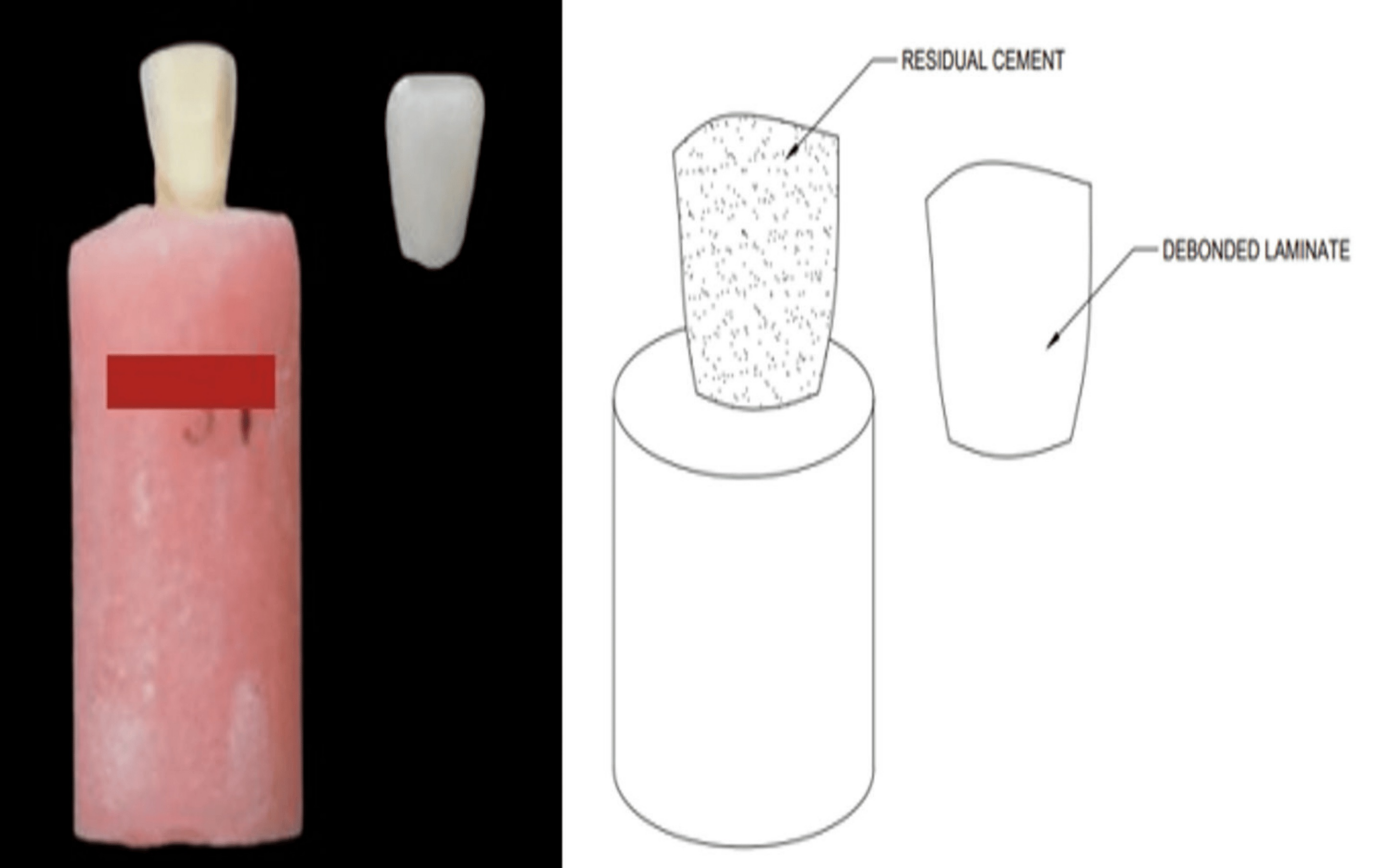
Cement interface fracture explained with a schematic diagram. Image credit: Pranjal S Soni

The veneered samples were positioned vertically, and a load was applied using a load platform placed at the center of the incisal edge. The 40 specimens were loaded at a crosshead speed of 0.5 mm/min until fracture of the veneers occurred. The maximum load at which the veneer separated from the prepared tooth was recorded, and fracture resistance was calculated. The values were noted for the fractured and debonded veneers. All the samples were scanned under a stereomicroscope at 40x magnification to evaluate the type of fracture. Specimens were examined for the type of failure: laminate fracture (Figure 3) and cement interface fracture (Figure 4).

## Results

**Figure 5:**
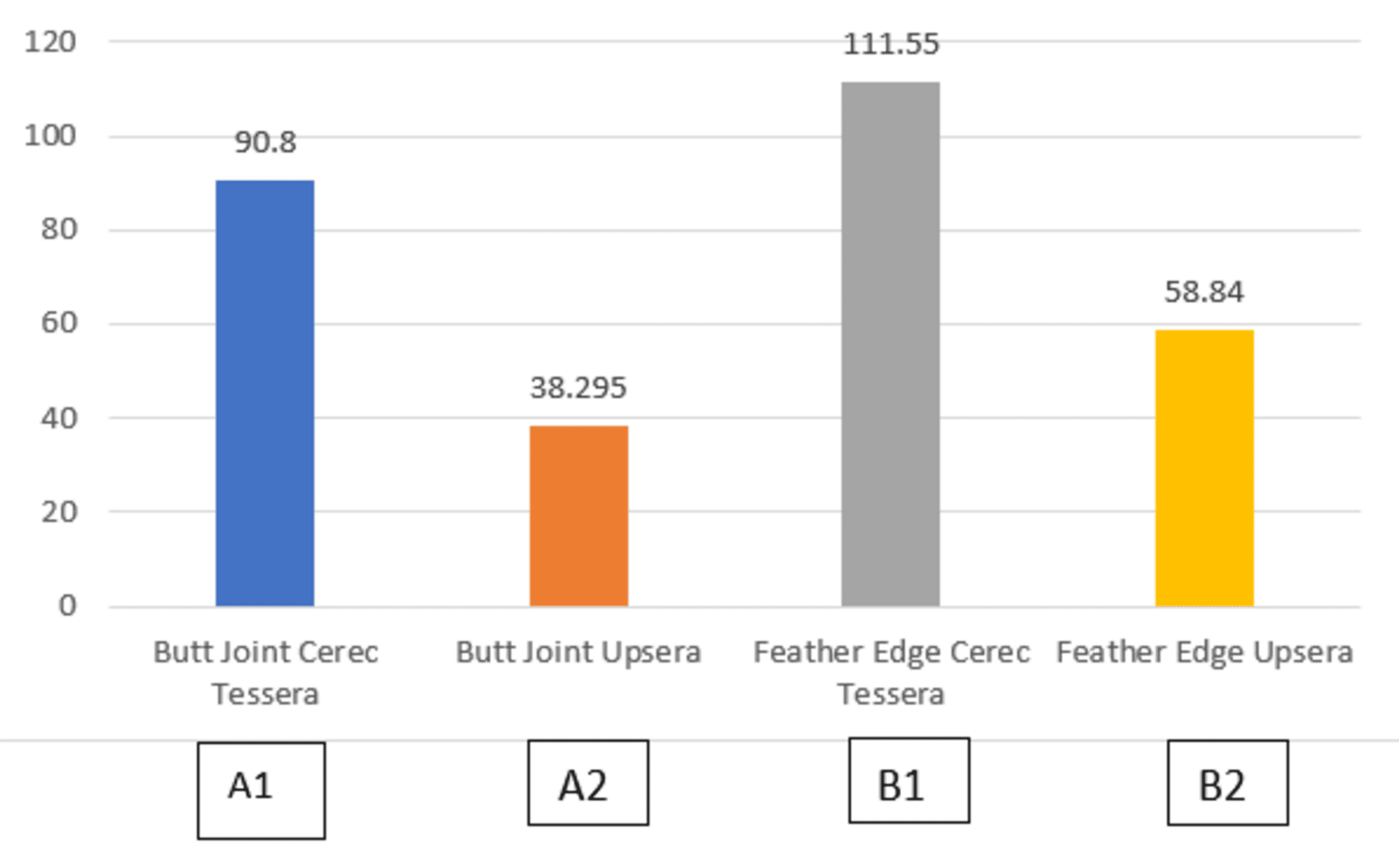
Comparison of fracture resistance between all four groups

A total of 40 samples were obtained and divided into two groups of 20 each based on the material used: Group 1 (Cerec Tessera) and Group 2 (Upcera). Each group consisted of two preparation designs: butt joint and feather edge, with 10 samples per preparation design. Descriptive statistics for fracture resistance were expressed as mean ± standard deviation (SD). An inter-group comparison was performed, where fracture resistance among the four groups was compared using a one-way ANOVA followed by a post hoc Tukey test. The mode of failure among the four groups was assessed using a chi-square test. The post hoc Tukey test was applied for pairwise comparisons. The data were subjected to statistical analysis at a 95% confidence level. In these tests, a p-value ≤ 0.05 was considered statistically significant. All analyses were performed using SPSS software version 26.0 (IBM Corp., Armonk, NY, USA). Table 1 and Figure 5 show the comparison of fracture resistance among the four groups.

**Table 1:** Comparison of fracture resistance between all four groups. N (Newton)

Group name	Number of Samples	Mean	Std. Deviation	F Value	P Value
Butt Joint Cerec Tessera (A1)	10	90.80 N	5.78	413.90	0.00 HS
Butt Joint Upcera (A2)	10	38.29 N	4.16		
Feather Edge Cerec Tessera (B1)	10	111.55 N	5.82		
Feather Edge (B2)	10	58.84 N	4.25		

The mean for Butt Joint Cerec Tessera (A1) was 90.80 N with a standard deviation of 5.79; the mean for Butt Joint Upcera (A2) was 38.30 N with a standard deviation of 4.16; the mean for Feather Edge Cerec Tessera (B1) was 111.55 N with a standard deviation of 5.82; and the mean for Feather Edge Upcera (B2) was 58.84 N with a standard deviation of 4.26. Table 2 shows that a highly significant F value of 413.90 (p < 0.01) was obtained from the ANOVA, suggesting significant variations in fracture resistance among the four groups. A highly significant difference was observed between all pairs, with a p-value less than 0.01 (p < 0.01), and a standard deviation of 2.3 according to the post hoc Tukey test.

**Table 2:** Determination of the type of failure after fracture resistance testing

Group		Type of Failure		Chi square	p value
		Cement Interface Fracture	Laminate Fracture		
Butt Joint Cerec Tessera (A1)	Count	5	5	8.57	0.03 S
	% within Group	50.0%	50.0%		
Butt Joint Upcera (A2)	Count	2	8		
	% within Group	20.0%	80.0%		
Feather Edge Cerec Tessera (B1)	Count	5	5		
	% within Group	50.0%	50.0%		
Feather Edge Upcera (B2)	Count	0	10		
	% within Group	0.0%	100.0%		

**Figure 6:**
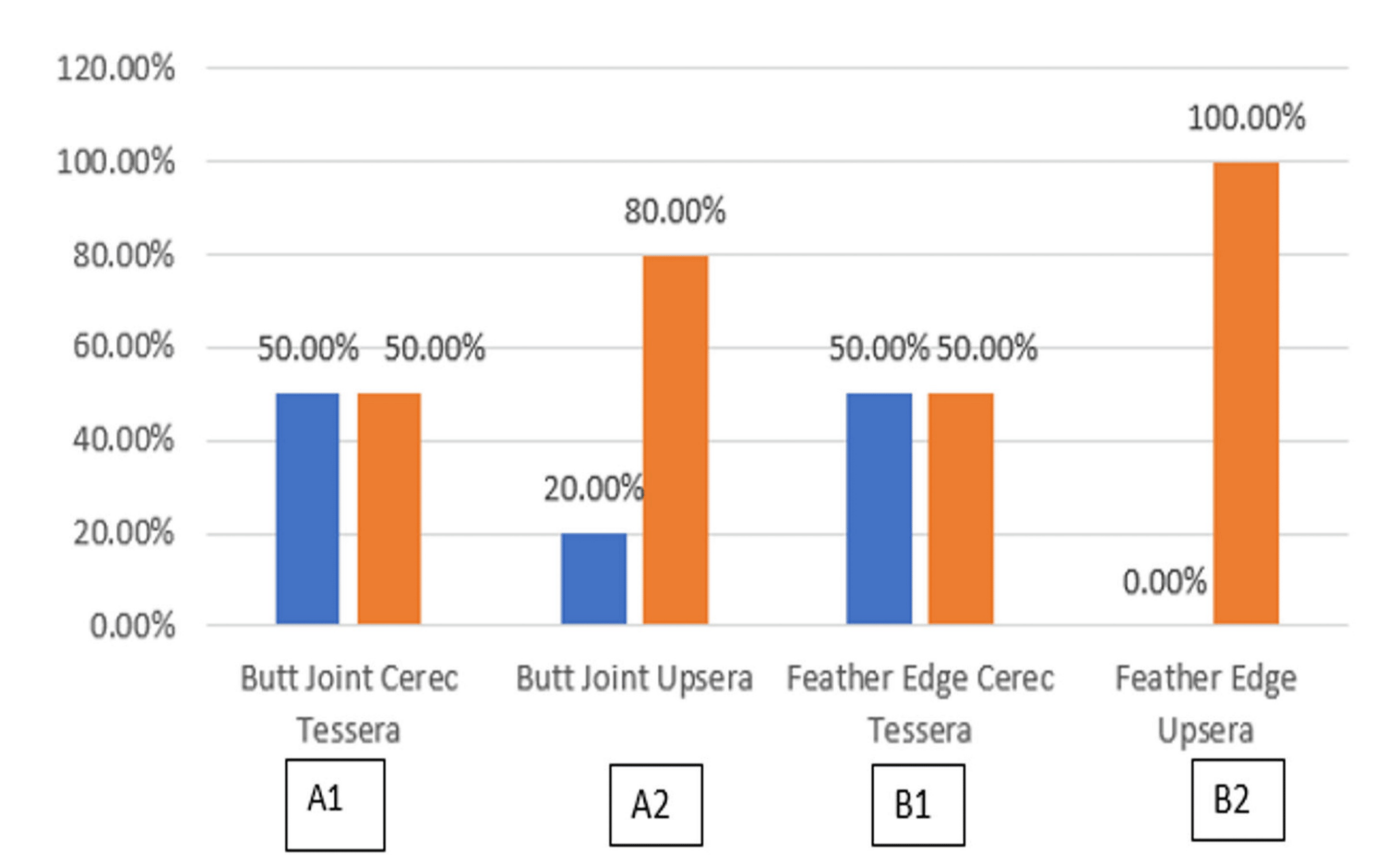
Determination of the type of failure after fracture resistance testing Blue bars: cement interface fracture, Orange bars: laminate fracture

The distribution of modes of failure among the four groups is shown below (Figure 6). Within the Butt Joint Cerec Tessera group (A1), there were five cases of cement interface fracture and five cases of laminate fracture, each accounting for 50.0%. In contrast, the Butt Joint Upcera group (A2) had two cases of cement interface fracture (20.0%) and eight cases of laminate fracture (80.0%). The Feather Edge Cerec Tessera group (B1) had an equal distribution of five cases each for both types of failure (50.0% each), while the Feather Edge Upcera group (B2) had no cases of cement interface fracture (0.0%) but had 10 cases of laminate fracture (100.0%).

## Discussion

Veneer restorations were traditionally cemented on unprepared tooth surfaces. Recent guidelines advise cautious intraenamel preparations with a gingival chamfer measuring 0.3-0.5 mm [4]. There is variance in preparation design concerning the incisal edge. Some clinicians recommend preserving the incisal edge, while others favor overlying it. Butt joint preparation involves incisal reduction of less than or equal to 2 mm with a 90-degree lingual marginal finish line. The preparation extends to the interproximal area, keeping the contact area intact [5]. The benefits of incisal butt preparation include the veneer's desirable character at the incisal third, achieved by a flat incisal wall and incisal reduction. Additionally, the enamel coating around the periphery is preserved. The butt joint design reduces the possibility of shrinkage-related post-insertion cracks and offers a favorable ceramic/luting ratio [6].

The materials chosen to restore the laminate veneer restorations were Cerec Tessera and Upcera. Cerec Tessera is an advanced lithium disilicate ceramic made of lithium disilicate and virgilite, a lithium aluminum silicate. Upcera is a lithium disilicate glass-ceramic known for its high strength due to its microstructure, which features randomly small and interlocking plate-like crystals. These materials were selected due to their popularity among clinicians. The laminate veneers were cemented using the routine cementation protocol and then subjected to thermocycling. Thermocycling replicates the thermal changes that occur in the oral cavity by repeatedly exposing restorative materials to hot and cold temperatures in water baths. This process simulates the aging of the materials in vivo. According to Gale and Darvell's 1999 hypothesis, 10,000 heat cycles are equivalent to one year's worth of clinical function [7]. The samples were kept in the thermocycling apparatus in water baths held at 5°C to 55°C with a dwell time of 30 seconds each for 10,000 cycles prior to fracture resistance testing. The fracture resistance of the veneers was evaluated using a universal testing machine. The specimens were loaded at a crosshead speed of 0.5 mm/min until fracture of the veneers occurred. The anticipated survival rate for teeth with feathered incisal edge preparation is at least 80% at 1.5, seven, and 11 years in situ, according to follow-up data on the clinical performance of ceramic veneers. Investigations ranging from 1.5 to seven years have shown that survival rates for preparations with butt joints are greater than 92% [8]. The results from the fracture resistance tests indicated that the preparation design and the choice of ceramic material influence the fracture resistance of veneers. Cerec Tessera with feather edge preparation yielded the maximum fracture resistance, while Upcera with butt joint preparation showed the least fracture resistance.

A number of variables affect how fractures or veneer debonding occur, including the restoration's shape, thickness, length, microstructural features, and elastic modulus of the ceramic material; mistakes made during the clinical stages of the work; surface flaws or exposed dentin; errors during the technical manufacturing of the restoration; and the strength and direction of the force [9]. The bite force of humans can vary widely depending on factors such as age, individual strength, and dental health. On average, humans can exert a bite force ranging from around 70 to 125 Newtons (N) [10], or even higher in some cases. Parafunctional habits, such as teeth clenching, bruxism, or nail biting, can generate significant force on the teeth and surrounding structures. The exact amount of force generated can vary widely depending on the individual and the intensity of the habit. Studies have shown that during bruxism episodes, forces ranging from 100 to 300 Newtons (N), or even higher, can be exerted on the teeth [11]. During normal chewing, incisors may experience forces ranging from 20 to 70 Newtons (N). The most frequent reason for veneer restoration failure is fracture [12]. Patients with poorly aligned teeth, parafunctional habits, and cementation to pre-existing restorations are associated with higher fracture rates. Static, cohesive, and adhesive are the three main types of veneer failures documented. Static fractures occur when a veneer segment breaks but remains whole. The restoration may be overloaded, or the resin cement may shrink during polymerization, leading to static fractures. Failure can be directly attributed to the amount of unsupported porcelain and the internal fit of the ceramic restoration. Static fractures can be avoided by minimizing internal stresses with a fit discrepancy of 100 μm or less. Cohesive fractures are caused by the tensile strains that excessive functional or parafunctional loading places on the porcelain restoration. Similar to how a metal coping for a metal-ceramic crown provides rigidity, enamel gives the tooth rigidity. Cohesive fractures within the porcelain body might directly impact the stress-strain distribution of the veneer when the enamel is removed. Under any force, the resulting flexure can cause a cohesive fracture. Because any lack of adhesion might increase stress upon load, maintaining enamel at the cervical and incisal regions is crucial. 

The bonding interface between the cement, porcelain restoration, and tooth is directly responsible for adhesive fractures. At least 86% of adhesive fractures occur at the cement/dentin interface, suggesting that either a poor bond or excessive occlusal load may be to blame. There were zero cases of crown fractures in the study. The feather edge design with Cerec Tessera demonstrated the highest fracture resistance, indicating its potential as a favorable combination for achieving optimal veneer durability. After the tests, the samples were observed for the type of failure, and the study identified laminate fractures as the most common type of fracture observed in the veneer restorations. Cement interface fractures ranked highest in frequency, indicating that this type of fracture occurred most often under the tested conditions. The study's limitations include its in vitro design, which may not fully capture the complexities of the oral environment in vivo. Variables such as saliva, fluctuating oral temperatures, and patient-specific conditions could affect the outcomes in a clinical setting. Although the study suggests that feather edge Cerec Tessera restorations might be suitable for patients with bruxism, further research is needed to assess the long-term performance of these restorations in such cases.

## Conclusions

Feather edge preparation with Cerec Tessera laminate veneer restoration demonstrated the highest fracture resistance, followed by butt joint preparation with Cerec Tessera laminate veneer restoration. Feather edge preparation and butt joint preparation, when combined with Upcera laminate veneer restoration, showed significantly lower fracture resistance compared to Cerec Tessera laminate veneer restoration. Feather edge preparation with Upcera laminate veneer restoration showed the highest incidence of laminate fractures, followed by butt joint preparation with Upcera laminate veneer restoration. Cement interface fractures were equivalent for both butt joint and feather edge preparations restored with Cerec Tessera laminate veneer restoration. The study identified laminate fractures as the most common type of fracture observed in the veneer restorations, followed by cement interface fractures.
